# Sensitivity and specificity of monoclonal and polyclonal immunohistochemical staining for West Nile virus in various organs from American crows (*Corvus brachyrhynchos*)

**DOI:** 10.1186/1471-2334-7-49

**Published:** 2007-05-30

**Authors:** Rebecca C Smedley, Jon S Patterson, RoseAnn Miller, Jeffrey P Massey, Annabel G Wise, Roger K Maes, Ping Wu, John B Kaneene, Matti Kiupel

**Affiliations:** 1 Diagnostic Center for Population and Animal Health and Department of Pathobiology and Diagnostic Investigation, MI, USA; 2 Center for Comparative Epidemiology, College of Veterinary Medicine, Michigan State University, East Lansing, MI, USA; 3 Molecular Biology Section, Michigan Department of Community Health, Lansing, MI, USA

## Abstract

**Background:**

Based on results of earlier studies, brain, heart and kidney are most commonly used for West Nile virus (WNV) detection in avian species. Both monoclonal and polyclonal antibodies have been used for the immunohistochemical diagnosis of WNV in these species. Thus far, no studies have been performed to compare the sensitivity and specificity of monoclonal and polyclonal antibodies in detecting WNV in American crows (*Corvus brachyrhynchos*). Our objectives were to determine 1) the comparative sensitivities of monoclonal and polyclonal antibodies for immunohistochemical (IHC) diagnosis of WNV infection in free-ranging American crows, 2) which organ(s) is/are most suitable for IHC-based diagnosis of WNV, and 3) how real-time RT-PCR on RNA extracted from formalin-fixed paraffin-embedded tissues compared to IHC for the diagnosis of WNV infection.

**Methods:**

Various combinations, depending on tissue availability, of sections of heart, kidney, brain, liver, lung, spleen, and small intestine from 85 free-ranging American crows were stained using a rabbit-polyclonal anti-WNV antibody as well as a monoclonal antibody directed against an epitope on Domain III of the E protein of WNV. The staining intensity and the extent of staining were determined for each organ using both antibodies. Real-time RT-PCR on formalin-fixed paraffin-embedded tissues from all 85 crows was performed.

**Results:**

Forty-three crows were IHC-positive in at least one of the examined organs with the polyclonal antibody, and of these, only 31 were positive when IHC was performed with the monoclonal antibody. Real-time RT-PCR amplified WNV-specific sequences from tissue extracts of the same 43 crows that were IHC-positive using the polyclonal antibody. All other 42 crows tested negative for WNV with real-time PCR and IHC staining. Both antibodies had a test specificity of 100% when compared to PCR results. The test sensitivity of monoclonal antibody-based IHC staining was only 72%, compared to 100% when using the polyclonal antibody.

**Conclusion:**

The most sensitive, readily identified, positively staining organs for IHC are the kidney, liver, lung, spleen, and small intestine. Real-time RT-PCR and IHC staining using a polyclonal antibody on sections of these tissues are highly sensitive diagnostic tests for the detection of WNV in formalin-fixed tissues of American crows.

## Background

West Nile virus (WNV) first emerged in the Western hemisphere during the 1999 New York City outbreak and has since spread across the United States, into Canada, and to the Caribbean Islands and Central America [1-6]. This virus is expected to spread throughout South America in the next few years [7]. The New York WNV strain is closely related to the virulent WN-Israel 1998 virus strain isolated from a goose [8], which may explain the surprisingly high number of avian fatalities, especially among American crows (*Corvus brachyrhynchos)*, following the 1999 New York City outbreak [1,4]. The North American WNV epizootic caused fatal disease in more than 200 avian species as well as in reptilian, and mammalian species, including humans [1,4,9-16]. Mortality rates in some avian species, such as corvids, can approach 100% [17]. These unusually high mortality rates may be due to the introduction of WNV in naïve avian populations, or due to the emergence of a new virulent strain [1-4].

The identification of WNV-positive birds has been shown to be the earliest indicator of WNV in an area [18]. American crows (AMCRs) are the most sensitive sentinel species used to detect the presence of WNV in northern regions [19-21]. However, in other regions, different species have been shown to be more sensitive than the crow, such as the blue jay in the Southern United States [21,22]. A study by the Centers for Disease Control and Prevention (CDC), using data from 2002, found that in 379 of 527 counties (72%) reporting human West Nile meningoencephalitis, the first reported human cases occurred a median of 38.5 days after the first WNV-affected dead bird had been found [23]. Corvids infected with WNV are usually found dead without any previously reported clinical signs, or die within 24 hours of the onset of clinical signs. Because of the acute onset and rapidly progressive nature of the disease, significant gross and histologic lesions are rarely observed at necropsy [4,24]. Therefore, appropriate tissue collection and diagnostic testing are imperative for accurate diagnosis and usefulness in a WNV surveillance program.

To date, few studies have been performed to determine appropriate tissue selection, test sensitivity and test specificity for WNV surveillance. Earlier studies to determine the best tissue(s) for WNV detection in AMCRs using RT-PCR and virus isolation (VI) reported that virus was most consistently detected in fresh samples of kidney and brain [4,24,25]. In addition, the heart, lung, liver, kidneys, and spleen were determined to be good organs for VI and RNA detection [4,24]. Immunohistochemistry (IHC) has also been documented as a reliable and efficient method of identifying WNV in formalin-fixed avian tissues [4]. One study reported that IHC using a polyclonal antibody was comparable to VI for the detection of WNV in birds [26]. In another report, sections of heart, kidney, and spleen were consistently positive using, VI, RT-PCR, and a polyclonal antibody for IHC staining [4]. As a result of these studies and recommendations by the CDC, the brain, heart and kidneys are used most commonly for WNV detection in avian species [21]. While new molecular WNV surveillance tests have been developed in recent years, many veterinary diagnostic laboratories still use immunohistochemistry as a primary method for WNV surveillance in AMCRs.

The rabbit-polyclonal anti-WNV antibody, that has been used for IHC staining in most studies,, is not specific for WNV, and can detect other flaviviruses (including St. Louis encephalitis virus) [4]. In contrast, a commercially available monoclonal anti-WNV antibody (7H2) has been shown to be specific for the detection of WNV. One study, using this antibody for IHC staining, reported that sections of heart and kidney were just as reliable for the diagnosis of WNV infection as RT-PCR performed on fresh brain tissue in AMCRs. Other tissues (bone marrow, duodenum, proventriculus, liver, lung, spleen, pancreas, and brain) used for IHC staining were not as reliable [27]. In another recent study, using the same monoclonal antibody, it was reported that kidney and spleen were more often positive than brain and skin in infected birds; however, none of the tissues could consistently identify a positive bird and the authors did not recommend the use of this monoclonal antibody for immunohistochemical WNV surveillance [28]. Thus far, no studies have been performed to directly compare the sensitivity and specificity of a monoclonal antibody to a polyclonal antibody for detection of WNV in AMCRs.

The objectives of this study were to determine: 1) if there is a significant difference in IHC staining for WNV using monoclonal versus polyclonal antibodies in naturally WNV-infected, free-ranging AMCRs, 2) which organ(s) is/are the most appropriate for the detection of WNV infection by these IHC techniques, based upon sensitivity and staining intensity, and 3) how real-time RT-PCR performed on RNA extracted from formalin-fixed paraffin-embedded tissues compared to IHC for the diagnosis of WNV infection. Only IHC-positive crows that were confirmed WNV-positive by RT-PCR were considered, in order to rule out cross-reaction with other flaviviruses. Because of WNV's wide host range and continued spread to new regions, ongoing assessment of surveillance techniques is needed in order to generate accurate, useful data for predicting the occurrence of human WNV infections.

## Methods

Eighty five free-ranging AMCRs that had been found dead in the State of Michigan during the summer of 2001, and had originally been submitted for WNV surveillance, were used in this study.

### Immunohistochemistry

IHC examination was performed for each of these 85 AMCRs using various combinations of sections of heart (79), kidney (73), brain (54), liver (50), lung (42), spleen (39), and small intestine (54). The sections chosen for evaluation depended upon tissue availability for each bird. Formalin-fixed tissues were trimmed, embedded in paraffin, sectioned at 5 μm and routinely processed for immunohistochemistry. An Enhanced V Red (Alkaline Phosphatase Red) Detection System, (Ventana Medical Systems, Inc., Tucson, AZ, USA) as well as bulk buffers specifically designed for use on the BenchMark Automated Staining System, (Ventana Medical Systems, Inc., Tucson, AZ, USA) were used for immuno-labeling and visualization. Slides were baked in a drying oven at 60°C for 30 minutes. The slides were then barcode labeled, and placed in the BenchMark for deparaffinization and heat-induced epitope retrieval. Antigen retrieval was performed using retrieval solution CC1 (Medical Systems, Inc., Tucson, AZ, USA) with an 8-minute heating and an 8-minute cooling cycle. A rabbit-polyclonal anti-WNV antibody (BioReliance, Rockville, MD, USA) at a concentration of 1:2000 for 32 minutes, and, a commercially available mouse-monoclonal anti-WNV antibody (clone 7H2), directed against an epitope on Domain III of the E protein of WNV (BioReliance, Rockville, MD, USA), at a concentration of 1:2000 for 32 minutes were used as primary antibodies. The slides were counterstained using Ventana hematoxylin (Ventana Medical Systems, Inc., Tucson, AZ, USA), and bluing for 2 minutes each, then dehydrated, cleared and mounted. Formalin-fixed, paraffin-embedded sections of heart and kidney from crows that had previously been tested for WNV infection using RT-PCR were used as positive and negative controls.

### Evaluation of immunohistochemical staining

Two board-certified pathologists (JSP and MK) independently reviewed all slides and identified each available organ as either positive or negative by monoclonal and/or polyclonal antibody staining. If at least one organ was positive, the crow was determined to be positive for WNV by IHC. In addition, each organ was evaluated for focal versus diffuse staining, and the type(s) of cells that exhibited positive staining in each organ was/were recorded. All IHC-positive (monoclonal and/or polyclonal) organs were subjectively evaluated for the percentage of positive cells within an organ and the intensity of antibody staining. A value of 1, 2, or 3 was assigned to each IHC-positive organ based on the approximate percentage of positive-staining cells (including inflammatory cells) in each organ. A value of 1 was assigned if less than 20% of the cells within an organ stained positively; a value of 2 was assigned if 20% to 80% of the cells within an organ stained positively; a value of 3 was assigned if greater than 80% of the cells within an organ stained positively. In addition, to determine IHC staining intensity, each organ was assigned a value of 1, 2, or 3 based on the ease of identifying positive staining at a magnification of 2.5× on a light microscope. A value of 1 was assigned if positive staining could not be identified at 2.5× (but was identified at higher magnification); a value of 2 was assigned if faint positive staining could be identified at 2.5×; a value of 3 was assigned if positive staining was easily identified at 2.5×. Average scores for each organ, in each category, were determined.

### RT-PCR

#### Frozen tissue samples

Fresh frozen, pooled tissue samples from all IHC-positive crows and 5 selected IHC-negative crows were submitted to the Molecular Biology Section of the Michigan Department of Community Health (MDCH) Laboratory, Lansing, MI, for RT-PCR detection of WNV as previously published [10,29]. RT-PCR was used as the gold standard to confirm WNV infection in IHC-positive crows.

#### Formalin-fixed, paraffin-embedded tissue samples

At the time of data evaluation, fresh frozen tissues were no longer available for any of the crows. Therefore, in order to check for viral RNA in the tissues of the IHC negative crows, SYBR Green-based Real-time RT-PCR was performed on pooled, paraffin-embedded tissues from all 85 crows by the Virology Section of the Diagnostic Center for Population and Animal Health at Michigan State University. Prior to RNA extraction, approximately 25 mg of tissue were deparaffinized in 1 ml of Citrisolv (Fisher Scientific International, Hampton, NH, USA), then washed twice in 1 ml of 100% ethanol and air-dried for 10 minutes. Total RNA was extracted using the RNeasy Mini Kit (QIAGEN, Inc., Valencia, CA, USA) and eluted in 50 μl of RNAse-free water. RT-PCR was performed with an IQ5 real-time PCR detection system (Bio-Rad Laboratories, Hercules, CA, USA) using the Quantitect SYBR Green RT-PCR Kit (QIAGEN, Inc., Valencia, CA, USA). The primers used for SYBR Green-based Real-time RT-PCR, 5'-TCGGGTCATTTGAAGTGTAGAGT-3' (forward) and 5'-CCATCCGTGCCAGTGTACTGC-3' (reverse), amplify a 155-bp region of the envelope glycoprotein E gene of WNV. Primers were at a 0.5 μM concentration in a 50 μl reaction volume. Five μl of the extracted RNA was used as template. Cycling conditions were: 50°C for 30 min, 95°C for 15 min, followed by 45 cycles of 94°C for 10 sec, 55°C for 30 sec and 72°C for 10 sec. A post-amplification melt curve analysis was done to detect WNV-specific products with an approximate peak melting temperature of 80.5°C.

### Statistical analysis

The sensitivity and specificity of both the monoclonal and the polyclonal antibody staining in comparison to the RT-PCR (using formalin-fixed, paraffin-embedded tissues) results were determined for specific organ samples. Differences in results between tests were assessed using Fisher's Exact test. In addition to sensitivity and specificity, the positive and negative predictive values were calculated for the monoclonal and the polyclonal antibody staining for specific organ samples.

## Results

### IHC and evaluation of immunohistochemical staining

Forty-three out of 85 crows showed IHC-positive staining, in at least one of the examined organs, with the polyclonal antibody and 31 of these 43 were IHC-positive with both monoclonal and polyclonal techniques. No birds were positive with only monoclonal antibody staining. In these 43 IHC-positive cases 97.5% (39/40) of the kidneys, 93.3% (14/15) of the spleens and only 35.3% (6/17) of the brains examined were positive with the polyclonal antibody. The heart, liver, lung, and small intestine were positive in 100% (42/42, 15/15, 13/13, 18/18 respectively) of the cases. Using the monoclonal antibody, 47.6% (20/42) of the hearts, 62.5% (25/40) of the kidneys, 66.7% (10/15) of the spleens, 29.4% (5/17) of the brains, 66.7% (10/15) of the livers, 76.9% (10/13) of the lungs, and 83.3% (15/18) of the small intestines examined were positive. Both pathologists scored all tissues identically.

**Figure 1:**
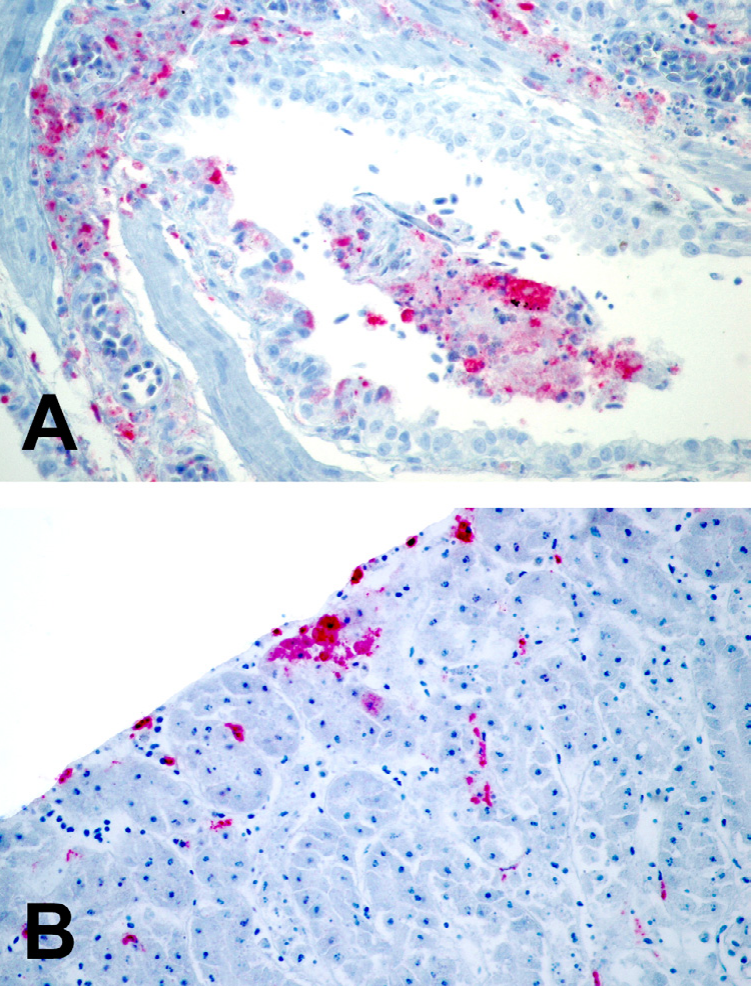
Intense, multifocal, red immunohistochemical staining for West Nile virus in the lung (A) and the kidney (B) using the polyclonal antibody.

**Figure 2:**
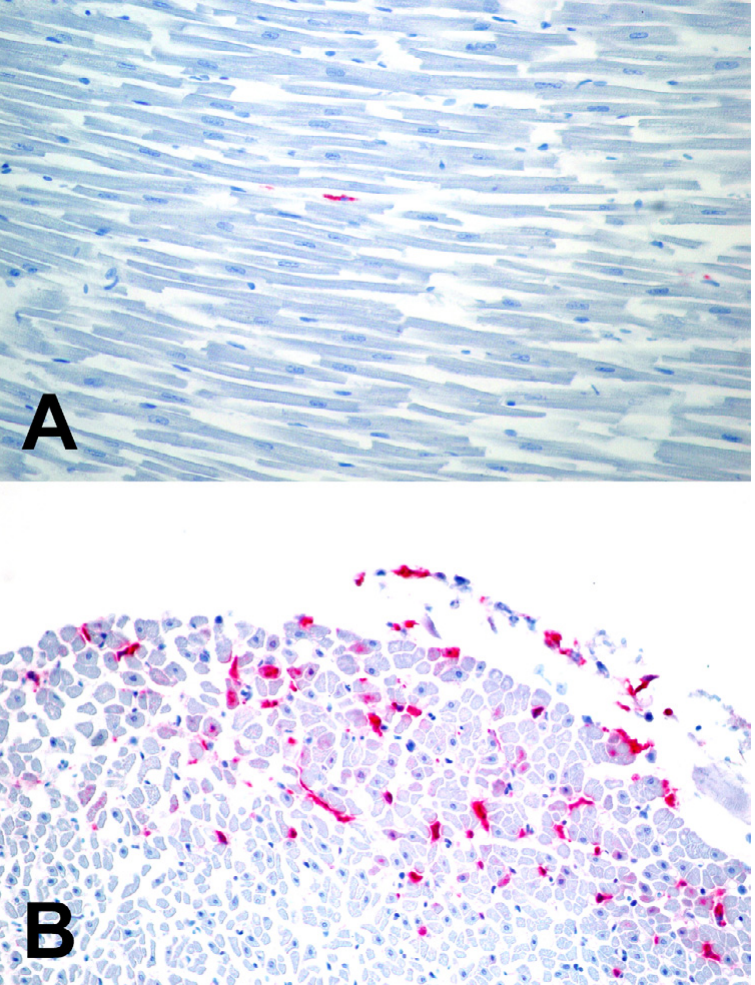
Pale, focal, red immunohistochemical staining for West Nile virus in the myocardium (A) using the polyclonal antibody; Intense, multifocal, red immunohistochemical staining for West Nile virus in the endocardium (B) using the polyclonal antibody.

The pattern of staining, and the individual cell types which stained positively, were also determined. Positive staining for WNV was always intracytoplasmic. The lung and kidneys exhibited intense multifocal staining (figure 1) and the liver, spleen, and small intestine exhibited intense multifocal to diffuse staining. The heart most commonly exhibited pale focal to diffuse staining, with the exception of some sections exhibiting intense epicardial and/or endocardial staining (figure 2). The brain exhibited rare focal staining of individual neurons and glial cells. Resident macrophages and intravascular blood monocytes were the most common positive-staining cells in all organs examined. This finding agrees with Weingartl's study in experimentally-infected crows and supports the hypothesis that monocytes/macrophages are target cells that help to spread the virus throughout many tissues [30]. Within sections of kidney, there were multifocal areas of positive staining within interstitial stromal cells, mononuclear inflammatory cells, and occasional tubular and collecting duct epithelial cells. Staining in lung sections was often patchy and appeared to surround airways and vessels. Interstitial fibroblasts, intravascular and interstitial mononuclear inflammatory cells, sloughed bronchial epithelial cells, and occasional intact bronchial epithelial cells exhibited positive staining. Within sections of liver and spleen, the most commonly staining cells were Kupffer cells and reticuloendothelial cells. Staining of hepatocytes and splenic lymphocytes was not noted. Within sections of small intestine, staining of epithelial cells was intense and ranged from multifocal (individual crypts and occasional villi) to diffuse (all crypts and villi). Sections of heart usually exhibited pale scattered staining of interstitial connective tissue cells, interstitial mononuclear inflammatory cells and myofibers. Occasional endothelial cells also showed positive staining. Some sections exhibited intense staining of macrophages within the epicardium and endocardium. Staining within sections of brain was often confined to neurons and glial cells in focal areas. Occasional sections exhibited staining within the meninges (perivascular connective tissue cells and interstitial and intravascular mononuclear inflammatory cells). These findings are similar to other reports of cellular IHC staining for WNV in birds [4,22,25-27,30].

The numbers of organs assigned a specific score, based upon the approximate percentage of positive staining cells (including inflammatory cells), and the ease of positive stain identification, using the polyclonal and the monoclonal antibody, are presented in Table 1. Average scores for each organ in each category are presented in Table 2. Based on these results, the organs that exhibited the highest percentage of positive staining cells with both antibodies were the spleen, liver, lung, and small intestine. The organs with the highest staining intensity (in which staining was easily identified at 2.5×), with both the monoclonal and the polyclonal antibody, were the same organs that exhibited multifocal or diffuse staining, and include the small intestine, spleen, lung, liver, and kidney.

**Table 1:** Numbers of samples classified by ease of identification (staining intensity) and percentage of positive staining cells, by tissue type and IHC staining method

Tissue	Staining Method	Number of samples classified by ease of identification at 2.5×			Number of samples classified by percentage of positive cells		
		Unable	Identifiable	Easy	< 20%	20–80%	> 80%
Heart	Monoclonal	10	9	1	20	0	0
	Polyclonal	17	24	1	33	9	0
Kidney	Monoclonal	1	16	8	23	2	0
	Polyclonal	4	28	7	32	7	0
Brain	Monoclonal	2	3	0	5	0	0
	Polyclonal	5	1	0	6	0	0
Spleen	Monoclonal	1	3	6	3	7	0
	Polyclonal	0	6	8	2	12	0
Liver	Monoclonal	1	7	2	6	4	0
	Polyclonal	2	6	7	3	12	0
Lung	Monoclonal	0	3	7	7	3	0
	Polyclonal	0	7	6	8	5	0
Small Intestine	Monoclonal	0	5	10	8	4	3
	Polyclonal	0	1	17	5	8	5

**Table 2:** Average value assigned to each organ for staining intensity (ease of identification) and percentage of positive staining cells by tissue type and IHC staining method

	Monoclonal Antibody		Polyclonal Antibody	
	Ease of identification at 2.5×^a^	Percentage of positive cells^b^	Ease of identification at 2.5×^a^	Percentage of positive cells^b^

Heart	1.55	1.00	1.62	1.21
Kidney	2.28	1.08	2.08	1.18
Brain	1.60	1.00	1.17	1.00
Spleen	2.50	1.70	2.57	1.86
Liver	2.10	1.40	2.33	1.80
Lung	2.70	1.30	2.46	1.38
Small intestine	2.67	1.67	2.94	2.00

^a ^Ease of identification:

1 = positive staining could not be identified at 2.5× (but was identified at higher magnification); 2 = faint positive staining could be identified at 2.5×; 3 = positive staining was easily identified at 2.5×

^b ^Percentage of positive cells:

1 = less than 20% of the cells stained positively; 2 = 20% to 80% of the cells stained positively; 3 = greater than 80% of the cells stained positively

### RT-PCR

#### Frozen tissue samples tested by Taqman-based real-time RT-PCR

WNV RNA was detected in fresh frozen, pooled tissues from all 43 of the IHC-positive crows by TaqMan RT-PCR assay and was not detected in any of the 5 IHC negative cases for a total of 43 confirmed WNV positive crows.

#### Formalin-fixed, Paraffin-embedded tissue samples tested by SYBR Green-based real-time RT-PCR

WNV RNA was detected in pooled, paraffin-embedded tissues from all 43 of the IHC positive crows using this method; this finding is consistent with the RT-PCR results using pooled frozen tissue samples. WNV RNA was not detected in any of the 42 IHC negative crows. These results validated the use of formalin-fixed, paraffin-embedded tissue samples for RT-PCR detection of WNV RNA.

### Statistical analysis

Both monoclonal and polyclonal antibodies had a staining specificity of 100% when compared to PCR results (Table 3). The overall sensitivity of monoclonal IHC staining was 72% (31 monoclonal positive crows/43 PCR positive crows) and the sensitivity of polyclonal antibody staining was 100%. The sensitivity of polyclonal antibody staining was higher than monoclonal antibody staining for all specific tissues tested, and 100% sensitivity was achieved with polyclonal antibody staining of heart, liver, lung, and small intestine samples. The sensitivity of polyclonal antibody staining was statistically significantly (p ≤ 0.05) higher than monoclonal antibody staining for kidney, brain, and small intestine tissue samples (Table 3).

**Table 3:** Sensitivity and specificity of antibody staining techniques (monoclonal and polyclonal IHC) for the detection of WNV in avian tissue samples, using RT-PCR as a gold standard (n = 85, 43 positive, 42 negative by RT-PCR)

		# of Samples			Sensitivity		Specificity	
		All	Pos	Neg	Pt. Est.	95% C.I.	Pt. Est.	95% C.I.
Overall*	Monoclonal	85	31	54	72.1	57.3 – 83.3	100	91.6 – 100
	Polyclonal	85	43	42	100	91.8 – 100	100	91.6 – 100
Heart	Monoclonal	79	20	59	47.6	33.4 – 62.3	100	90.6 – 100
	Polyclonal	79	42	37	100	91.6 – 100	100	90.6 – 100
Kidney*	Monoclonal	73	25	48	62.5	47.0 – 75.8	100	89.6 – 100
	Polyclonal	73	39	34	97.5	87.1 – 99.6	100	89.6 – 100
Brain*	Monoclonal	54	5	49	29.4	13.3 – 53.1	100	90.6 – 100
	Polyclonal	54	6	48	35.3	17.3 – 58.7	100	90.6 – 100
Spleen	Monoclonal	38	10	28	66.7	41.7 – 84.8	100	85.7 – 100
	Polyclonal	37	14	23	93.3	70.2 – 98.8	100	85.1 – 100
Liver	Monoclonal	50	10	40	66.7	41.7 – 84.8	100	90.1 – 100
	Polyclonal	50	15	35	100	79.6 – 100	100	90.1 – 100
Lung	Monoclonal	40	10	30	76.9	49.7 – 91.8	100	87.5 – 100
	Polyclonal	42	13	29	100	77.2 – 100	100	88.3 – 100
Small Intestine	Monoclonal	50	15	35	83.3	60.8 – 94.2	100	89.3 – 100
	Polyclonal	53	18	35	100.0	82.4 – 100	100.0	90.1 – 100

* Indicates statistically significant difference between monoclonal and polyclonal tests, Fisher's Exact p ≤ 0.05

**Figure 3:**
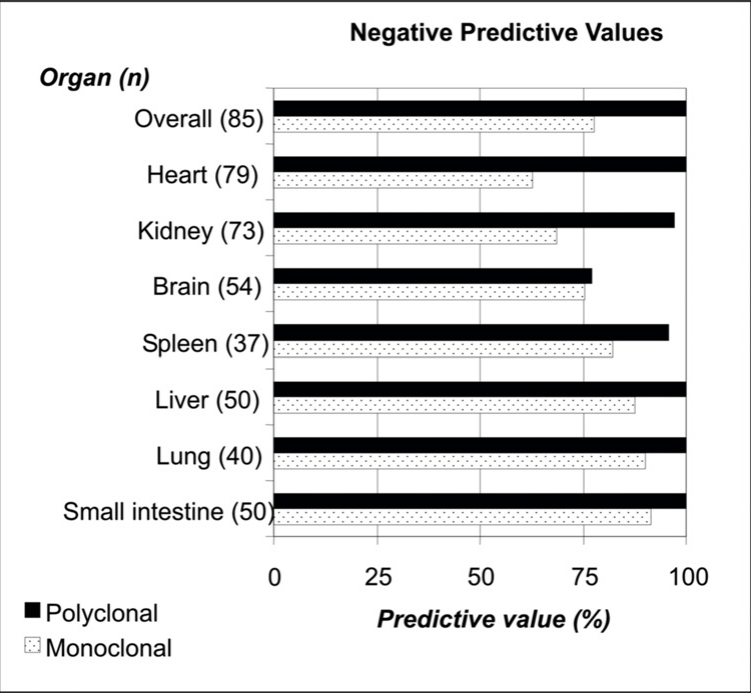
Negative predictive values of IHC antibody staining of specific tissues for WNV, using RT-PCR as a gold standard.

The positive predictive values for both monoclonal and polyclonal IHC staining were 100%, regardless of sample type. Negative predictive values of polyclonal IHC staining were higher than those for monoclonal staining (figure 3). Interestingly, the difference in negative predictive values for monoclonal and polyclonal staining of brain tissues was relatively small (75.5% versus 77.1%, respectively). The best negative predictive values were seen for polyclonal antibody staining of the heart, liver, lungs, and small intestine (100%).

## Discussion

The test sensitivity of IHC staining for the detection of WNV infection in AMCRs using a polyclonal antibody with the Benchmark IHC System is superior to that of the 7H2 monoclonal antibody. When compared to RT-PCR results, the sensitivity of monoclonal IHC staining was 72%, and the sensitivity of polyclonal IHC staining was 100%. Therefore, the monoclonal antibody is not recommended as a screening/surveillance test for WNV in AMCRs. The polyclonal antibody was also significantly more sensitive than the monoclonal antibody in detecting WNV infection in each organ tested. However, it is important to note that the polyclonal antibody has been shown to detect other flaviviruses, whereas the monoclonal antibody does not. Even though the polyclonal antibody is the superior antibody for WNV surveillance in AMCRs, its use may be limited in geographical regions that harbor other flaviviruses, and additional tests may be required to exclude those viruses.

An interesting finding was the low percentage of IHC positive hearts using the monoclonal antibody (47.6%) compared to the polyclonal antibody (100%). This difference may be due to the relatively small amount of antigen observed within heart sections. In spite of the high percentage of IHC-positive heart sections stained with the polyclonal antibody, the percentage of positive cells in sections of liver, lung, spleen, and small intestine was greater than that seen in the heart. This correlates with the finding that sections of kidney, lung, liver, spleen and small intestine were far superior to sections of heart and brain for the identification of positive WNV staining at low magnification (2.5×) using both antibodies. The intensity of staining depended, in part, upon the pattern of IHC staining exhibited by each organ. Organs that exhibited intense multifocal staining and intense diffuse staining were most easily identified as IHC-positive at low magnification (2.5×). Based on the sensitivity of each organ, the intensity of staining, and the pattern of staining, sections of kidney, spleen, liver, lung and small intestine were shown to be superior to the heart and brain for IHC staining for WNV in AMCRs. These results correlate with Weingartl's findings in experimentally infected crows [30]. Another important finding in this study was that the RT-PCR results using formalin-fixed, paraffin-embedded tissues showed 100% correlation with the results of the RT-PCR performed on pooled fresh tissue from 48 birds (43 IHC-positive and 5 IHC-negative birds). The risk of human exposure to WNV during testing and processing of tissues is decreased by performing RT-PCR on formalin-fixed tissues. (Similar to other RNA extraction profiles, biosafety concerns can also be addressed by immediately placing fresh tissues into a lysis buffer for RNA extraction.)

The monoclonal antibody, clone 7H2, is specific for an epitope on Domain III of the envelope (E) protein [31,32]. This epitope is most likely a conformational epitope, and therefore, monoclonal antibody sensitivity may be affected by heat retrieval, formalin fixation and autolysis. The epitopes targeted by the polyclonal antibody are unknown, but most likely include both conformational and linear epitopes on multiple structural proteins. This could explain the significantly lower sensitivity of the monoclonal antibody compared to the polyclonal antibody in this study.

Mosquito-borne transmission is believed to be the most common form of WNV infection in birds; however, other routes may also play a role. Our finding of consistent, intense IHC staining for WNV in sections of small intestine and lung supports the possibility of oral transmission, as well as gastrointestinal and respiratory shedding, of the virus in free-ranging AMCRs, as suggested by others [11,17,30,33-35].

## Conclusion

Dead bird surveillance will continue to be an important tool for the early detection of WNV in a given area and for guiding control efforts [19,20]. In North America, where crows have been determined to be the most sensitive sentinel species for detecting WNV, sections of kidney, liver, lung, spleen and small intestine should be sampled for IHC testing. Because individual organs can show focal staining, multiple organs run simultaneously will yield the most accurate IHC results. Polyclonal antibody IHC staining should be used for WNV detection in AMCRs because its sensitivity is comparable to that of RT-PCR using both fresh and formalin-fixed tissues, as opposed to monoclonal antibody IHC staining which has a significantly lower sensitivity. RT-PCR can accurately be performed using formalin-fixed, paraffin-embedded tissue sections. In conclusion, we found that, provided appropriate tissues are selected, both polyclonal antibody-based IHC and RT-PCR on formalin-fixed tissues are excellent methods for the diagnosis of WNV in AMCRs.

## Competing interests

The author(s) declare that they have no competing interests.

## Authors' contributions

RCS evaluated the pattern, extent, and intensity of immunohistochemical staining in each sample and drafted the manuscript. JSP and MK initially interpreted the immunohistochemical staining for each sample and helped to draft the manuscript. RA and JBK performed the statistical analysis. JPM oversaw the RT-PCR performed on fresh frozen tissue samples. AGW, RKM, and PW performed the real-time RT-PCR on formalin-fixed paraffin-embedded tissue samples. All authors read and approved the final manuscript.

## Pre-publication history

The pre-publication history for this paper can be accessed here:


